# Atopic dermatitis-derived *Staphylococcus aureus* strains: what makes them special in the interplay with the host

**DOI:** 10.3389/fcimb.2023.1194254

**Published:** 2023-06-14

**Authors:** Antonietta Lucia Conte, Francesca Brunetti, Massimiliano Marazzato, Catia Longhi, Linda Maurizi, Giammarco Raponi, Anna Teresa Palamara, Sara Grassi, Maria Pia Conte

**Affiliations:** ^1^ Department of Public Health and Infectious Diseases, Sapienza University of Rome, Rome, Italy; ^2^ Department of Infectious Diseases, Istituto Superiore di Sanità, Rome, Italy; ^3^ Department of Public Health and Infectious Diseases, Sapienza University of Rome, Laboratory Affiliated to Institute Pasteur Italia- Cenci Bolognetti Foundation, Rome, Italy; ^4^ Dermatology Clinic, Department of Clinical Internal, Anesthesiological and Cardiovascular Sciences, Sapienza University of Rome, Rome, Italy

**Keywords:** atopic dermatitis, *Staphylococcus aureus*, whole-genome sequencing, in silico genotype, pan-genome, biofilm, antibiotic resistance

## Abstract

**Background:**

Atopic dermatitis (AD) is a chronic inflammatory skin condition whose pathogenesis involves genetic predisposition, epidermal barrier dysfunction, alterations in the immune responses and microbial dysbiosis. Clinical studies have shown a link between *Staphylococcus aureus* and the pathogenesis of AD, although the origins and genetic diversity of *S. aureus* colonizing patients with AD is poorly understood. The aim of the study was to investigate if specific clones might be associated with the disease.

**Methods:**

WGS analyses were performed on 38 *S. aureus* strains, deriving from AD patients and healthy carriers. Genotypes (i.e. MLST, *spa-*, *agr-* and SCC*mec*-typing), genomic content (e.g. virulome and resistome), and the pan-genome structure of strains have been investigated. Phenotypic analyses were performed to determine the antibiotic susceptibility, the biofilm production and the invasiveness within the investigated *S. aureus* population.

**Results:**

Strains isolated from AD patients revealed a high degree of genetic heterogeneity and a shared set of virulence factors and antimicrobial resistance genes, suggesting that no genotype and genomic content are uniquely associated with AD. The same strains were characterized by a lower variability in terms of gene content, indicating that the inflammatory conditions could exert a selective pressure leading to the optimization of the gene repertoire. Furthermore, genes related to specific mechanisms, like post-translational modification, protein turnover and chaperones as well as intracellular trafficking, secretion and vesicular transport, were significantly more enriched in AD strains. Phenotypic analysis revealed that all of our AD strains were strong or moderate biofilm producers, while less than half showed invasive capabilities.

**Conclusions:**

We conclude that in AD skin, the functional role played by *S. aureus* may depend on differential gene expression patterns and/or on post-translational modification mechanisms rather than being associated with peculiar genetic features.

## 1 Introduction

Atopic dermatitis (AD) is a chronic inflammatory skin disease, characterized by intense itching and recurrent eczematous lesions, that affect 15-20% of children and 2-5% of adults in industrialized countries (Sybilski et al., 2015; Hadi et al., 2021). It usually occurs during early childhood with onset in 45% of cases within the first six months of the child’s life and 60% within the first year of life (Weidinger et al., 2018). There are many contributors to the pathogenesis of AD, including genetic defects in the epidermal barrier that can be explained, at least in part, by inherited mutations in keratinocyte proteins such as filaggrin and loricrin (Kim et al., 2008; Howell et al., 2009; Callewaert et al., 2020), an imbalance towards a T-helper-2 (Th2) immune response in which the cytokines IL-4, IL-5 and IL-13 play a major role (Homey et al., 2006; Brandt and Sivaprasad, 2011), and microbial dysbiosis that increases the susceptibility to AD (Bjerre et al., 2017). Numerous studies have shown that over 90% of patients with AD have *Staphylococcus aureus* skin colonization (Bieber, 2008; Kong et al., 2012; Totté et al., 2016; Meylan et al., 2017; Altunbulakli et al., 2018).

*S. aureus* is an important human pathogen in both community and hospital settings but it can also establish a commensal relationship with humans. The anterior nares represent the main habitat for *S. aureus*, which persistently colonized 20–30% of the population (Peacock et al., 2001; van Belkum et al., 2009). Metagenomic analysis has shown that the disease activity is significantly associated with the dysbiosis of skin microbiota, which is characterized by a reduced microbial diversity during eczema flare, allowing the proliferation of *S. aureus* (Kong et al., 2012). The most colonized sites are the lesional skin (56–96.2%), the nose (46.1–64.1%), and the non-lesional skin (28–39%). Interestingly, 65–77.3% of AD patients were colonized both in the anterior nares and on the skin, whereas only 10.2% of healthy control subjects were colonized on the skin (Na et al., 2012; Totté et al., 2016; Alsterholm et al., 2017; Clausen et al., 2017). Moreover, some reports found that 55% of AD patients were persistent carriers of *S. aureus* (Alsterholm et al., 2017). However, its role in the pathogenesis of AD remains poorly understood. *S. aureus* employs multiple mechanisms to colonize the skin. They can produce adhesins, such as i) fibronectin-binding proteins (FnBP) A and B, expressed on the surface to promote the bacterial adherence to fibronectin (Cho et al., 2001), ii) the cell-wall-anchored (CWA) protein ClfB, that binds to the cornified envelope proteins loricrin and cytokeratin (Leonard et al., 2019), iii) the CWA protein IsdA, that renders the bacteria much more resistant to the inhibitory effects of the fatty acids, a property that may contribute to colonization of AD skin (Clarke et al., 2007), and iv) the *S. aureus* Protein A (spa), involved in pro-inflammatory responses (Gómez et al., 2004). Recently, it has been evidenced a predominance of *S. aureus* strains forming biofilms in AD lesions and this has been directly related to disease severity (Di Domenico et al., 2018). Biofilm represents a survival strategy protecting bacteria from antimicrobials and immune-mediated clearance (Brandwein et al., 2016). *S. aureus* expresses a plethora of secreted virulence factors (VF) that contribute to the epidermal barrier disruption, such as the phenol-soluble modulins (PSMs) peptides, that includes the PMSα and PMSβ families along with the δ-toxin. These factors are highly cytotoxic to a wide variety of cells (e.g. keratinocytes), contribute to the biofilm development (which is important for staphylococcal colonization and persistence) and trigger the skin inflammation upon the epidermal colonization (Peschel and Otto, 2013; Nakagawa et al., 2017). PSMs are regulated by the quorum sensing system, which is called Accessory Gene Regulator (*agr*) (Le and Otto, 2015). This regulation is essential for the timing of VFs expression during infection (Nakamura et al., 2020). Superantigens, such as the staphylococcal enterotoxin (SE) SEA, SEB, SEC, SED, and toxic shock syndrome toxin-1 (TSST-1) were the most common toxin genes carried by *S. aureus* isolated from AD patients (Kim et al., 2009). TSST-1, SEA, and SEB activate polyclonal T cells and subsequently cause T cell-mediated inflammation in AD lesions (Park et al., 2016). About 34% of the patients were colonized by α-toxin-producing *S. aureus*. The pore-forming α-toxin is likely to play an important role in disrupting the skin barrier by inducing keratinocyte cell death and Th2 cytokine production (Breuer et al., 2005). *S. aureus* also produces extracellular proteases that contribute to primary sensitization to allergens, abrogating the epidermal permeability barrier (Hirasawa et al., 2010). In recent years, it has become evident that *S. aureus* is a facultative intracellular pathogen, able to invade and survive in a range of cell types. Major invasion factors include the fibronectin-binding proteins (FnBPs), staphylococcal autolysin (Atl), and the lipoprotein-like (*lpl*) gene cluster (Jevon et al., 1999; Sinha and Fraunholz, 2010; Nguyen et al., 2015; Huitema et al., 2021). Intracellular localization, protecting *S. aureus* from the immune system and most antimicrobial treatments, is associated with chronic or relapsing staphylococcal infections (Horn et al., 2018). Moreover, among *S. aureus* strains colonizing AD patients, the percentage of methicillin-resistant *S. aureus* is 4–13 times higher than in a healthy population; however, methicillin-resistant *S. aureus* isolates seems to be much lower in adult patients as compared to the children (Kim et al., 2009; Lo et al., 2010; Pascolini et al., 2011; Rojo et al., 2014). Typing of *S. aureus* at the genetic level by multi-locus sequence typing (MLST) revealed that ST188, ST1, ST5, and ST513 were the most frequent STs identified in AD patients. Furthermore, clonal complexes (CCs) (based on the *spa-*type) showed that CC1 was associated with AD patients and was more prevalent in those carrying mutations in the gene encoding filaggrin (FLG) (Clausen et al., 2017), whereas CC30 was more common in healthy subjects (Clausen et al., 2019). However, the distribution of STs and staphylococcal CCs in AD patients points to significant heterogeneity, and no specific clone/clones prevailed in this group of patients. Conventional AD treatments involve emollients (medical moisturizers) and topical anti-inflammatory corticosteroids and calcineurin inhibitors (Carr, 2013). A plethora of pathogenesis-based treatments have been developed, including monoclonal antibodies that neutralize IgE or block specific interleukins or their receptors. The most advanced therapy is the IL-4 receptor blocker dupilumab which inhibits the function of IL-4 and IL-13, essential mediators of the Th2 pathway (Callewaert et al., 2020). In this study, we conducted a genotypic and phenotypic characterization of *S. aureus* isolated from AD patients during eczema flares and in the post-flare recovery phase, after treatment with dupixent^®^ (dupilumab), to deepen our current knowledge about the features of the *S. aureus* strains associated with the disease.

## 2 Materials and methods

Fifteen (7 females and 8 males) patients, mean age of 30.2 ± 8.9 years old (range: 20-45), were enrolled at the Clinic of Dermatology, Department of Clinical Internal, Anesthesiologic and Cardiovascular Sciences, “Sapienza” University of Rome from September 2019 to February 2021.

The inclusion criteria to the study were the following: i) Caucasian Ethnicity; ii) age between 20 and 45 years old; iii) moderate-to-severe disease severity assessed through the Scoring Atopic Dermatitis “score index” (SCORAD); iv) eligibility for treatment with dupixent^®^ (dupilumab).

The exclusion criteria were: i) local/systemic antibiotics, immunomodulatory or immunosuppressive therapies within 4 weeks prior to enrollment; ii) phototherapy treatments (nb-UVB, PUVA) within 4 weeks prior to enrollment; iii) participation to research trials with experimental treatments with other monoclonal antibodies; iv) past medical history of bone marrow transplant, intestinal diseases, diabetes mellitus or congenital immunodeficiencies.

### 2.1 Disease severity assessment and sample collection

At the time of enrollment, after signing the informed consent to the study, all the patients were subjected to clinical evaluation to assess the study eligibility and the degree of disease severity, according to the SCORAD index. The clinical diagnosis of AD was based on historical features, morphology and distribution of skin lesions, and associated clinical signs. The SCORAD index range lies between 0 and 103. Based on the SCORAD index results, AD patients have been classified into mild (≤25), moderate (26–50), and severe (≥ 51) forms.

Sampling was performed for each patient at two different stages of the disease: acute phase, characterized by an exacerbation of the disease on any skin region before starting treatment (t0); post-acute phase defined at least 4 weeks after reaching clinical remission with treatment (t1). All AD patients enrolled received dupixent^®^ (dupilumab) as per protocol (600 mg subcutaneous injection once, then 300 mg every 2 weeks). Twenty-four hours before all sampling time points, patients had to avoid washing and the use of topical emollients at the sampling skin site. A sterile plastic device consisting of the open end of a cylinder with a diameter of 8 cm was applied to the skin area to be sampled. All samples were taken at the level of the popliteal fossa, which is divided into two parts (lesional and non-lesional) and each area was rubbed for 30 s with a sterile swab moistened with 1x PBS. At the same time, sampling at the level of the nasal choana using a sterile swab moistened with 1x PBS was carried out.

### 2.2 *S. aureus* isolation, identification and collection

Skin swabs were placed in tubes containing 3 ml of tryptic soy broth (TSB, Oxoid, Italy), immediately capped, and shaken for 30 s using a vortex mixer to suspend the bacterial cells. A volume of 100 µl of each swab suspension was plated onto selective Columbia agar with 5% of sheep blood (CAP, Oxoid Italy), Mannitol Salt Agar (MSA, Oxoid Italy), and Tryptic Soy Agar (TSA, Oxoid Italy) plates. Nasal swabs were immediately plated on an elective and selective growth medium. After incubation, the bacterial colonies were identified and counted.

From those samples where *S. aureus* was detected, a single colony was amplified and stored in a maintenance freeze medium (15% glycerol) (Oxoid) at −80 °C until use. The bacterial load from infection sites was determined by the conventional standard plate count method. The bacterial identification was performed by MALDI-TOF (Matrix-Assisted Laser Desorption Ionization Time-Of-Flight, VITEK^®^ MS, BioMérieux) at the Laboratory of Microbiology Analysis of the “Policlinico Umberto I” Hospital of Rome. Seventeen *S. aureus* strains, previously isolated from the anterior nares of healthy carriers and part of the in-house culture collection, were used as control strains.

### 2.3 DNA extraction

*S. aureus* isolates from AD patients and healthy carriers were grown on TSA plates for 24 h at 37°C. Then, a single colony was inoculated in TSB and incubated for 18 h at 37°C. After incubation, 1.5 ml of each bacterial culture was centrifuged at 10000 rpm for 10 min.

DNA extraction on pelleted bacteria was conducted using “QIAamp DNA Mini Kit” (Qiagen, Germany), as the manufacturer’s instructions.

### 2.4 Random amplified polymorphic DNA-polymerase chain reaction

RAPD-PCR reactions were performed using the OLP 13 primer (5’-ACCGCCTGCT-3’) (Zare et al., 2019), and a 25μl reaction mix, containing 3mM MgCl2 (Bio Line, London, UK), 0.5 mM dNTPs (New England BioLabs, UK), 1 µM of primer, 1 U of Taq DNA polymerase (Bio Taq DNA polymerase, Meridian Bioscience, USA) in 1X PCR buffer (Meridian Bioscience, USA) and 3 μl of template dsDNA, was prepared. PCR was performed as follows: 1 cycle at 94°C for 5 min, followed by 40 cycles at 93°C for 1 min, 37°C for 1.5 min, and 72°C for 1 min, with a final extension at 72°C for 7 min (Mastercycler pro, Eppendorf, Germany). Amplification products were solved by electrophoresis on 1% agarose-TBE gel, stained with 5µl of “Midori Green Advance DNA Stain” (Nippon Genetics Europe GmbH, Germany), visualized under UV transilluminator (UVP Inc., Cambridge) and photographed with DigiDoc-It (UVP, Cambridge) system. To guarantee the reproducibility of RAPD-PCR profiles, the reactions were carried out in triplicate.

#### 2.4.1 RAPD-PCR profiles analysis

The DNA banding patterns of RAPD-PCR assays were analyzed using TotalLab TL120 Trace version 2006 (Nonlinear Dynamics). The Dice coefficient of similarity was calculated on the base of presence/absence of bands while the unweighted pair group method with arithmetic averages (UPGMA) as agglomerative method. Cluster analysis has been carried out by using the statistical excel plugin XLstat 7.5 (Addinsoft, USA). To distinguish clonally different groups, the similarity percentage cut-off was set at 80%.

### 2.5 Whole genome sequencing analysis

Whole Genome Sequencing (WGS) analysis were performed on the *S. aureus* genomes, which were isolated from both AD patients and healthy carriers and sequenced on the MiSeq platform, using the Nextera XT library preparation kit v3 according to the manufacturer’s instructions (Illumina, San Diego, CA, USA).

The quality of the short reads was evaluated using FastQc v. 0.11.5 (http://www.bioinformatics.babraham.ac.uk/projects/fastqc/), combined with MultiQC v.1.11 (Ewels et al., 2016). Using the Shovill wrapper (https://github.com/tseemann/shovill), the reads were trimmed by Trimmomatic v. 0.39 (Bolger et al., 2014) and then *de novo* assembled by SPAdes assembler v.3.15.3 (Bankevich et al., 2012). The open reading frames were predicted and annotated by Prokka v.1.14.6 (Seemann, 2014), using the dedicated databases for the *Staphylococcus* genus associated with the software.

The average Nucleotide Identity (ANI) analysis and the Ribosomal Multilocus Sequence Typing (rMLST) were performed using fastANI v. 1.32 (Jain et al., 2018) and querying the PubMLST dedicated database (Jolley et al., 2012), respectively, in order to verify the taxonomy assignment of sequenced strains.

A kmer search was performed by AgrVate v.1.0.1 (Raghuram et al., 2022) on the *agr*-group database associated with the software to determine the *agr* locus type of the isolated genomes. The *spa*-type was established using DNAGear (AL-Tam et al., 2012). The MLST was performed using MLST software (https://github.com/tseemann/mlst) v. 2.19.0, scanning the assembled genomes against the PubMLST scheme for *Staphylococcus aureus* (Jolley et al., 2018). Only exact matches were kept. Alleles characterized by a sequence coverage of 100% and a sequence identity lower than 100% were further investigated mapping the paired-end short reads of the isolate on the most similar reference alleles, using Bowtie2 v.2.3.5.1 (Langmead and Salzberg, 2012). The Staphylococcal cassette chromosome *mec* (SCC*mec*) typing was performed by querying the extended database of SCC*mec*Finder (Kaya et al., 2018), to characterize the methicillin-susceptibility of the isolates.

To determine the presence of genes associated to antibiotic resistance and VFs, the sequences were aligned against the Comprehensive Antibiotic Resistance Database (CARD) (Alcock et al., 2019), ResFinder (Bortolaia et al., 2020) and Virulence Factor Database (VFDB) (Chen et al., 2016), using Abricate (https://github.com/tseemann/abricate) v. 0.9.9. The identified genes were then filtered, setting a threshold of 80% for both sequence identity and sequence coverage.

A core-genome based phylogenetic tree was constructed using the Bacterial Pan Genome Analysis (BPGA) software package based on unweighted pair group method with arithmetic mean (UPGMA) and setting 80% as sequence identity cut-off (Chaudhari et al., 2016). In addition to our isolates, *S. aureus* USA300 (WGS Project accession: JADMJC000000000) was also included in the analysis while the *S. epidermidis* strain ATCC12228 (NCBI accession: NC_004461) was considered as the outgroup.

To investigate the presence of the *bsa* (bacteriocin of *S. aureus*) operon and the *dprA* (DNA processing protein A) gene, the reference sequences of such loci were extracted from the publicly available genomes of the *S. aureus* Newman strain (Genbank accession: AP009351) and the *S. aureus* N315 (Genbank accession: BA000018) strain, respectively, and aligned against the newly assembled *S. aureus* genomes, using the stand-alone version of the Blast algorithm v.2.12.0 (https://blast.ncbi.nlm.nih.gov/Blast.cgi).

A k-mer search by RUCS (Thomsen et al., 2017) was performed to identify unique and core genomic sequences associated with a single group of assemblies.

#### 2.5.1 R analysis

Data produced from the previous steps of the analysis were filtered, manipulated and tested for differences using R v.4.1.2 (https://www.R-project.org/) and its available libraries data.table (https://cran.r-project.org/package=data.table), reshape2 (Wickham, 2007) and dunn.test (https://cran.r-project.org/package=dunn.test). The results were then displayed using ggplot (https://ggplot2.tidyverse.org), ggtree (Yu et al., 2017), ggtext (https://cran.r-project.org/package=ggtext), ggpubr (https://cran.r-project.org/package=ggpubr) and gridExtra (https://cran.r-project.org/package=gridExtra).

Functions in the vegan package (https://cran.r-project.org/package=vegan) were applied on a binary matrix representing the gene clusters identified in each assembly, in order to compute a distance matrix and to perform a Principal Coordinate Analysis (PcoA); the Jaccard similarity index and the Lingoes correction for negative eigenvalues were applied to two steps of the analysis, respectively.

The Pan-genome composition was evaluated for each assembly and compared between the groups. A functional annotation was performed in order to assign a Cluster of Orthologous Groups (COG) category (https://www.ncbi.nlm.nih.gov/research/cog) to each gene cluster. The relative frequencies of the COG categories were computed for every component of the pan-genome.

### 2.6 Antibiotic susceptibility test

Antibiotic susceptibility profiles of *S. aureus* strains were defined by the guidelines of the European Committee on Antimicrobial Susceptibility Testing (EUCAST, v. 6.0) and performed by the automated microbial system Vitek-2 (BioMérieux), at the Laboratory of Microbiology Analysis of the Policlinico Umberto I, “Sapienza” University of Rome. The antibiotic susceptibility profile was defined by testing the following antibiotics: benzylpenicillin, oxacillin, gentamicin, levofloxacin, erythromycin, clindamycin, linezolid, daptomycin, teicoplanin, vancomycin, tetracycline, tigecycline, fusidic acid, mupirocin, rifampicin, trimethoprim/sulfamethoxazole.

### 2.7 Biofilm assay

*S. aureus* strains were tested for their ability to form biofilm as previously described (Stepanović et al., 2007). *S. aureus* ATCC 6538P was used as biofilm positive control.

Biofilms were quantified by measuring the absorbance at λ 570 nm (A570). The average optical density (OD) value was calculated and cut-off values (ODc) were established. ODc is defined as the average of OD of negative control + three standard deviations. According to their absorbance, isolates were defined as no biofilm producer OD ≤ ODc; weak biofilm producer ODc < OD ≤ (2 X ODc); moderate biofilm producer (2 X ODc) < OD ≤ (4 X ODc); strong biofilm producer (4ODc) < OD.

### 2.8 Cell line and culture conditions

The human immortalized keratinocyte cell line, HaCaT, was cultured in Dulbecco’s modified Eagle’s medium high glucose (DMEM, Corning, Italy) supplemented with 10% inactivated fetal bovine serum (FBS, Gibco, Italy), and 1% penicillin/streptomycin (Sigma-Aldrich, Italy) and maintained in 5% of CO2 atmosphere at 37°C.

### 2.9 Invasion assays

Invasion assays were performed in twenty-four- well culture plates seeded with HaCat cells (2x10^5^ cells/ml) and incubated at 37°C in 5% CO_2_ for 48 h. Cell monolayers were infected with bacterial suspensions (multiplicity of infection [MOI] of 10) in 0.5 ml of cell culture medium devoid of antibiotics. Bacteria were centrifuged onto cellular monolayers two times, each at 1200 rpm for 2.5 min, and incubated for 1 h at 37°C. After infection time, cell monolayers were washed, and incubated with fresh medium containing 200 μg/ml of gentamicin (Sigma-Aldrich, Italy) for 1 h, to kill extracellular bacteria. Finally, cells were lysed with Triton X-100 (0.1% v/v) and plated on TSA plates to determine the number of intracellular vital bacteria (calculated as cfu/ml).

Strains were considered invasive when the percentage of the ratio between the number of intracellular bacteria and the initial inoculum was ≥0.1%. Data represent the mean of three independent experiments in duplicate.

### 2.10 Statistical tests

An initial descriptive analysis was performed with tables and plots corresponding to the type of qualitative or quantitative variables. Statistical analysis was carried out using both parametric and non-parametric tests according to the analyzed variable. Particularly, non-parametric univariate statistical tests, X^2^ and Fisher’s test, were used for the discrete variables. For continuous variables, data were tested for normality using the Shapiro-Wilk Normality test, followed by the pairwise comparisons using T-test or Mann–Whitney U test. Comparisons among multiple groups were tested using Kruskal–Wallis test followed by pairwise Dunn’s *post hoc* tests. Levene’s test was used to assess the equality of variances of the groups.

The permutational multiple analysis of variance (PERMANOVA) test was calculated on distance matrices to assess the presence of statistically significant partitions between groups, by performing 1000 permutations.

The statistical association between ordinal and/continuous variables was assessed by Kendall’s rank correlation analysis.

When necessary, *p*-values were corrected, by using the Benjamin–Hochberg false discovery rate (FDR) procedure to account for multiple hypothesis testing. In each case, a *p*-value ≤ 0.05 was considered statistically significant.

## 3 Results

### 3.1 Patients’ characteristics and clinical response

Fifteen patients, were included in the study. At the time of the enrollment, 6 out of 15 patients were affected by a severe form of AD (SCORAD index≥75), while 9 out of 15 patients presented with a moderate form of AD (26 ≥ SCORAD index < 75). After the sampling, all the patients started a systemic treatment with dupilumab as per protocol (600 mg subcutaneous injection once, then 300 mg every 2 weeks). All the patients had a good response to treatment in an average time of 5.1 months with complete clinical remission of the disease in 5 out of 15 patients, while 9 out of 15 patients had a significant reduction of severity (SCORAD < 25). After the treatment, 3 of the patients with moderate AD at t0 showed a complete clinical remission, while 6 patients improved to mild AD. Concerning the severe cases of AD at t0, 2 patients showed a complete clinical remission while 4 improved to mild AD form. The only patient who didn’t show clinical remission was excluded from the study.

### 3.2 General colonization pattern

A total of 50 *S. aureus* isolates, of which 37 were from AD patients at t0 and 13 at t1, were obtained by microbiological analysis. *S. aureus* was detected at t0 i) both in the anterior nares and on the lesional skin in 80% (12/15) of the patients and ii) on all of the sampled anatomical sites in 40% (6/15), whereas iii) cutaneous colonization alone was found in 20% (3/15) of the patients.

At t1, 21.4% (3/14) of patients were nasally and skin colonized; 35.71% (5/14) showed *S. aureus* colonization only in the nose and 14.2% (2/14) only on the skin, while in four patients, *S. aureus* was completely eradicated.

To further examine the correlation between *S. aureus* skin colonization and AD severity, a quantitative detection of *S. aureus* was carried out. At t0, *S. aureus* was the predominant bacterial species on the lesional skin, whose bacterial load resulted in 10^4^- 10^6^ cfu/ml. Instead, a lower load of *S. aureus* (10 -10^2^ cfu/ml) was detected at t1, on the skin of AD patients. A significant positive correlation was found between the *S. aureus* load and disease status (severe, moderate, or mild) (τb =0.837; p-value < 0.0001).

### 3.3 A single *S. aureus* strain dominates in each AD patient

A RAPD-PCR analysis was carried out to determine the genetic relatedness of 50 *S. aureus* isolates from the skin and nose of 15 non-related AD patients. By using 80% similarity, a total of 21 distinctive clonal groups (strains) were detected (data not shown), 17 from AD patients at t0 and 4 from AD patients at t1. Furthermore, in 33.3% (5/15) of the patients, the t0 *S. aureus* isolates from the nose, the lesional skin and non lesional skin of the same subject belonged to the same clonal group; in 33.3% (5/15) of the patients, *S. aureus* isolates from the nose and the lesional skin of the same subject belonged to the same clonal group; in 13.3% (2/15) of the patients, *S. aureus* isolates from the lesional and non-lesional skin of the same subject belonged to the same clonal group; in 6.6% (1/15), *S. aureus* was recovered only in the lesional skin; in 6.6% (1/15) of the patients, isolates from nose and lesional skin showed no genetic relatedness and in 6.6% (1/15), the nasal and the lesional skin isolates belonged to the same clonal group, which was different from the one of the non lesional skin isolate.

At t1 *S. aureus* was recovered only from the nose of 57.1% (5/14) patients and these isolates resulted clonal to those recovered at t0 from the same patient. In 14.2% (2/14) patients, *S. aureus* was recovered at t1 only from the skin and these isolates were non-clonal with those isolated at t0 from the same patient. Both nasal and skin colonization at t1 was found in 21.4% (3/14) patients and the nasal isolates resulted clonal to those recovered at t0. Only in one patient, the t1 skin isolate was clonal to those recovered at t0. Overall, the examination of the intra-host genetic heterogeneity of the colonizing *S. aureus* showed a clonal expansion and diffusion of a single strain that can persist in the post-flare.

### 3.4 Whole genome sequencing analysis

WGS analysis was performed on 38 *S. aureus* strains (21 from AD patients and 17 from healthy carriers). Genomic assemblies obtained from high-quality short reads and their features are summarized in **Table S1**. The typical %GC content and genome size of *S. aureus* organisms, as well as the taxonomy assessment analysis supported the taxonomy assignment of the strains.

#### 3.4.1 *In silico* typing analysis

All *S. aureus* strains were typed according to MLST, *spa-*, *agr-* (**Table S2**), and SCC*mec*-typing.

##### 3.4.1.1 *spa*-typing

The *spa*-typing analysis evidenced the presence of 27 different types within the population, of which 3 were never reported before. No predominant types were observed, while the most prevalent ones were t1451, t091, and t0148 which were found in 13.1% (5/38), 10.5% (4/38), and 7.9% (3/38) of the population, respectively.

Among the AD-associated strains, 13 different *spa-t*ypes were identified, where t1451 and t091 were the two most prevalent types among the diseased subjects, each one characterizing 19% (4/21) of the strains. Likewise, both t359 and t008 were detected in 9.5% (2/21) of the AD-associated strains.

Concerning the healthy carrier strains, 14 different *spa*-types were identified and the most common types were t148 and t012, which were found in 17.6% (3/17) and 11.7% (2/17) of the strains, respectively. The latter *spa*-types (i.e. t4022, t153, t1994, t166, t331, t1078, t2777, t1509, t160, t449, t1451) were identified only once, composing up to 64.7% (11/17) of the group.

Three novel *spa*-types were identified. The AD strains 2pSAt0LS and 4pSAt0LS showed two novel *spa*-types, having similar repeat sequences to those of t449 and t148, respectively. A third novel *spa-*type was detected from the healthy carrier strain 29cSA, whose repeat sequence is similar to t2777. (**Table S3**)

No statistical differences were found in the distribution of *spa-*types between strains isolated from individuals with AD and those from healthy carriers.

##### 3.4.1.2 MLST

Analogously to the *spa*-typing, the MLST evidenced a high ST diversity due to the identification of 24 distinct types, of which 4 were never reported before. The most frequent type was ST7, which was observed in 13.1% (5/38) of the population. Among the 21 AD *S. aureus* strains, 12 different STs were identified. The group-wise evaluation showed that ST7, ST398, ST3510, ST5, and ST8 composed 23.8% (5/21), 9.5% (2/21), 9.5% (2/21), 9.5% (2/21), and 9.5% (2/21) of the AD strains, respectively. In three cases, *S. aureus* strains deriving from the same individual showed different STs. Furthermore, two AD strains isolated from the same patient were characterized by a novel *arcC* allele, whose sequence differs from allele 3 for a single C-T transition at position 303.

Among the *S. aureus* strains isolated from healthy carriers, 12 different STs were identified. In particular, ST72, ST30, ST34, and ST45 were observed in 17.6% (3/17), 11.7% (2/17), 11.7% (2/17), and 11.7% (2/17) of the strains, respectively. Two new alleles of *aroE* and *arcC* genes, respectively, were identified within the healthy carrier group. In particular, the first allele differed from *aroE* allele 5 with respect to a C-A transversion at position 420, while the second one differed from *arcC* 1 for a T-C transition at position 402. No statistically significant differences were observed in the distribution of STs between the two groups of strains.

##### 3.4.1.3 *agr*-typing

*agr*-I represented the most frequent allotype, which was found in 63.1% (24/38) of the strains. A lower prevalence was observed for the *agr*-II and *agr*-III, which composed 21% (8/38) and 13.1% (5/38) of the strains, respectively, while only one healthy carrier strain was classified as *agr-*IV. No statistically significant differences were found in the distribution of *agr*-types between strains from AD and healthy carriers.

##### 3.4.1.4 SCC*mec*-typing

According to the SCC*mec*-typing, the population is exclusively characterized by methicillin-susceptible *S. aureus* strains.

#### 3.4.2 Virulence factor genes

In the present study, the presence of VF genes was determined by aligning the genomes of our strains with the sequences present in the dedicated database VFDB.

Among the 100 VF genes identified in the whole population, 80 were found in ≥ 50% of the strains, as shown in **Figures S1**, **S2**. Virtually all VF genes associated with immune evasion, adhesion, and toxic activity (exoenzymes and toxins) were found in 100% of the population. By comparing the distribution of the VF coding sequences between the groups, no statistically significant differences have been evidenced, except for some enterotoxin genes (i.e. *sec, sei, sell, sem, sen, seo, and seu*) (**Figure S3**), that resulted significantly more prevalent in the healthy carriers group.

#### 3.4.3 Antimicrobial resistance genes identification

All the antimicrobial resistance (AMR) genes in the reference databases were tested for their presence and we noticed that each strain was characterized by at least *arlR, arlS, norA, mgrA, tet38, LmrS, mepA, mepR, cls, pgsA, mprF, walK, rpoB, fusA, gyrA, parC* and *parE*. NorA, Tet38, and LmrS efflux pumps are members of the major facilitator superfamily (MFS). NorA expression is regulated by the two-component system ArlRS and MgrA, and mediates the resistance to fluoroquinolones (e.g. ciprofloxacin). MgrA also regulates the expression of *tet38* gene (Truong-Bolduc et al., 2005), whose overexpression confers resistance to tetracycline (Chen and Hooper, 2018). Instead, the LmrS multidrug efflux pump is involved in the resistance mechanisms to aminoglycosides, macrolides, phenicols, diaminopyrimidine, and oxazolidinone (Floyd et al., 2010). Previous studies suggest that mutations on specific genes can mediate the resistance to tigecycline (*mepA* and *mepR*) (McAleese et al., 2005; Dabul et al., 2018; Fang et al., 2020), to daptomycin (*cls, pgsA, mprF*, and *walK*) (Peleg et al., 2012; Song et al., 2013), to rifampicin (*rpoB*) (Wichelhaus et al., 1999), to fusidic acid (*fusA*) (Lannergård et al., 2009), to trimethoprim/sulfamethoxazole (*dfrA* and *folP*) (Nurjadi et al., 2020) and to levofloxacin (*gyrA, parC* and *parE*)(Sanfilippo et al., 2011).

As shown in **Figure S4**, further genes were identified in some of our strains, although no statistically significant differences were detected between the groups. The *aac(6’)-Ie-aph(2’’)-Ia* and *ant(4’)-Ib* genes mediate the aminoglycoside resistance and they were detected among AD-associate strains in 4.76% (1/21) and in 9.52% (2/21), respectively, whereas only *ant(4’)-Ib* was identified among healthy carriers in 5.88% (1/17) of the strains.

*qacA* is another gene encoding for a MFS efflux pump and it confers resistance to several fluoroquinolones (Alam et al., 2003). This gene was found only in 5.88% (1/17) of AD-associated strains. The analysis revealed also the presence of AMR genes involved in the resistance to erythromycin (*ermC, ermT, mphC* and *msrA*), to clindamycin (*ermC* and *ermT*), to benzylpenicillin (*blaZ*), to fosfomycin (*fosB*) and to fusidic acid (*fusB*). Among the AD-associated strains, *ermT* was found in 19% (4/21), *mphC* in 9.52% (2/21), *msrA* in 9.5% (2/21), *blaZ* in 47.6% (10/21) and *fosB* in 42.8% (9/21). Instead, among the strains isolated from healthy carriers we detected *ermC* and *ermT* in 11.76% (2/17) and 5.88% (1/17), respectively. *fusB* was found in 5.88% (1/17), *msrA* in 5.88% (1/17), *mupA* in 5.88% (1/17), *blaZ* in 82.35% (14/17) and *fosB* in 58.8% (10/17).

##### 3.4.3.1 A wide antibiotic susceptibility characterizes all *S. aureus* strains

All 38 *S. aureus* strains underwent automated susceptibility testing with the Vitek-2 system using EUCAST breakpoints. As shown in **Table S4**, most of the strains were susceptible to a wide range of antibiotics, regardless of isolation origin. In particular, none of the *S. aureus* strains was resistant to gentamicin, linezolid, daptomycin, tetracycline, tigecycline, and rifampicin. Furthermore, no methicillin-resistant *S. aureus* strains were detected in both groups. Such finding agrees with the results of the *in silico* SCC*mec* typing. Resistance to benzylpenicillin is the most common AMR in the population, characterizing 68.42% (26/38) of strains, and it was more diffused among healthy carrier strains than AD ones (82% *vs* 57%). Both groups were also characterized by erythromycin-resistant strains, accounting for 26.31% (10/38) of the population, as well as clindamycin-resistant strains, accounting for 21.05% (8/38). Resistances to oxacillin and to teicoplanin were observed only among AD strains, in 10% (2/21) and in 5% (1/21) of strains, respectively. No statistically significant differences were found between the groups for any of the antibiotics.

It is worth noticing that multi-drug resistance was observed exclusively in the healthy carrier strain 12cSA (resistant to erythromycin, benzylpenicillin, mupirocin, clindamycin, and fusidic acid) and in the AD strain 14pSAt1S (resistant to erythromycin, benzylpenicillin, oxacillin, and trimethoprim/sulfamethoxazole).

An overall agreement between the WGS-predicted resistance and the automated phenotypic susceptibility testing was noticed in the bacterial population (**Table S5**). All strains were susceptible to gentamicin, linezolid, and vancomycin, whose corresponding AMR genes were not detected in the population. Instead, both the mupirocin- and fusidic acid-resistance genes were exclusively found in the *S. aureus* 12cSA strain, which was the only resistant strain to these antibiotics.

Concordance between the presence of at least one AMR gene associated with a specific antibiotic resistance mechanism and the corresponding resistant phenotype was observed for erythromycin, clindamycin, benzylpenicillin, oxacillin, and for teicoplanin, with some exceptions. For instance, i) 7pSAt0LS is the only benzylpenicillin susceptible-strain carrying the *blaZ* gene. ii) No corresponding AMR genes were found in some strains showing an intermediate or resistant phenotype to clindamycin [30% (3/10)], benzylpenicillin [7,70% (2/26)], erythromycin [9.09% (1/11)], teicoplanin [100% (1/1)] and oxacillin [100% (2/2)). iii] The automated phenotypic susceptibility testing revealed that all the strains were tetracycline-susceptible, although *tet38* and its regulator *mgrA* were ubiquitously found in the population.

In this study, we could not assess the concordance between genotypic and phenotypic profiles with respect to those antibiotics whose resistance is associated with a mutation on the specific genes (levofloxacin, rifampicin, tigecycline, daptomycin, and trimethoprim/sulfamethoxazole). Further studies will be conducted to investigate the presence of these genetic mutations.

#### 3.4.4 Core-genome phylogeny

**Figure 1:**
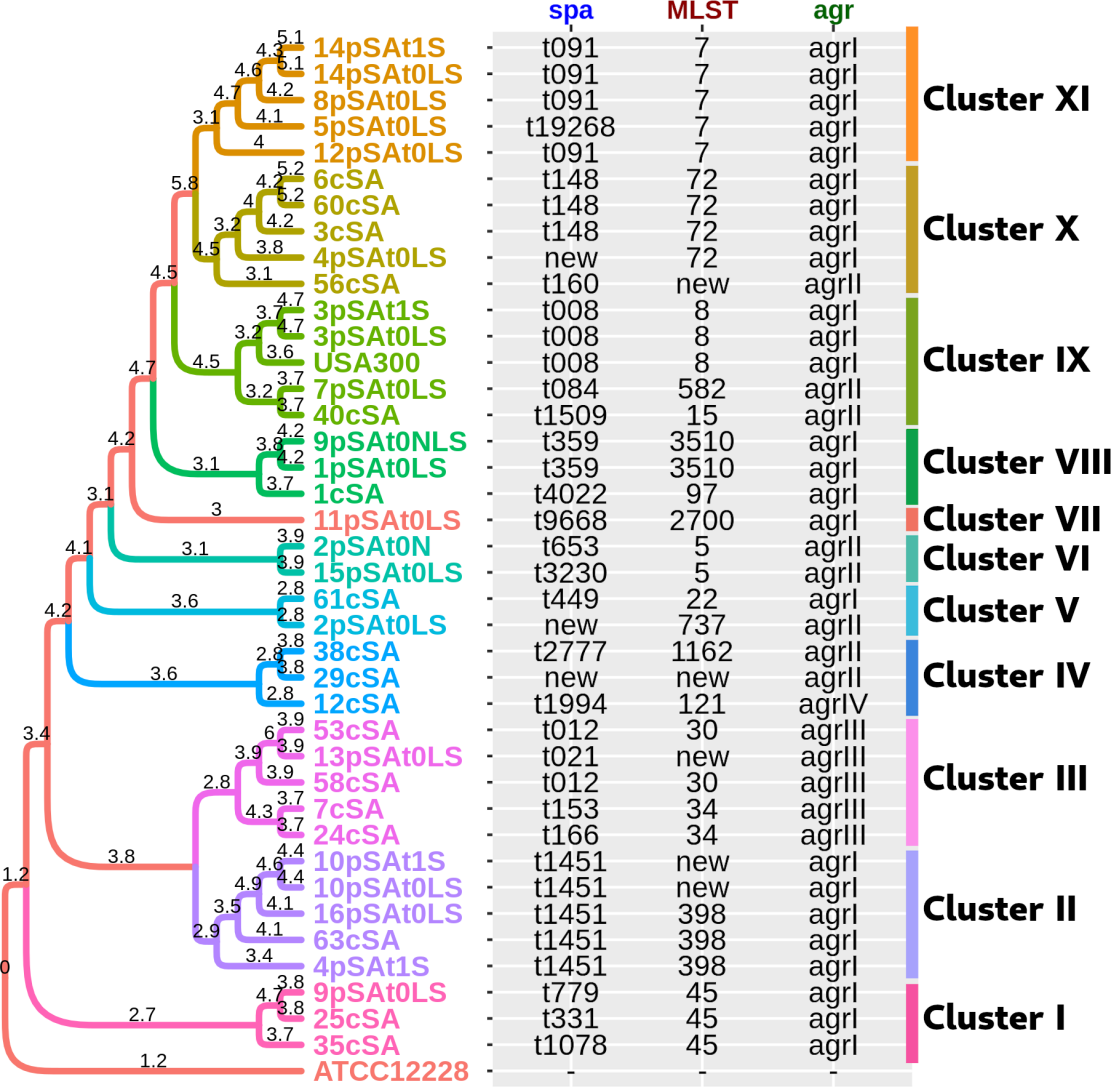
Core phylogenetic tree of the 38 *S. aureus* strains. The *S. aureus* USA300 strain was also included in the analysis, while the *S. epidermidis* strain ATCC12228, has been considered as the outgroup. Branch lengths (-log_10_ scale) expressed on the tree are proportional to the phylogenetic distances. Different colors were used to highlight the 11 clusters.

A phylogenetic reconstruction has been performed in order to investigate the genetic relationship among strains and to reveal a potential association with the presence of the disease. As shown in **Figure 1**, the population was split into 11 different clusters, each one characterized by strains sharing a similar *spa*, *agr*, and ST types as well as a similar set of VF and AMR genes. Furthermore, we observed that the strains deriving from the same patient tended to cluster together, although this was not the case for 4pSAt0LS and 4pSAt1S, 9pSAt0LS and 9pSAt0NLS, and 2pSAt0LS and 2pSAt0N. No statistically significant associations were observed between the identified clusters and the presence of the disease.

#### 3.4.5 Pangenomic analysis

The analysis of the pan-genome revealed 5939 groups of orthologous gene clusters, of which 2086 (52.69%) were present in all strains within the analyzed population (core-genome), 1468 (37.08%) were found in more than one strain but not in all (accessory-genome) while only a more limited number of OGC, 405 (10.23%), were identified in a single strain (unique-genome).

**Figure 2:**
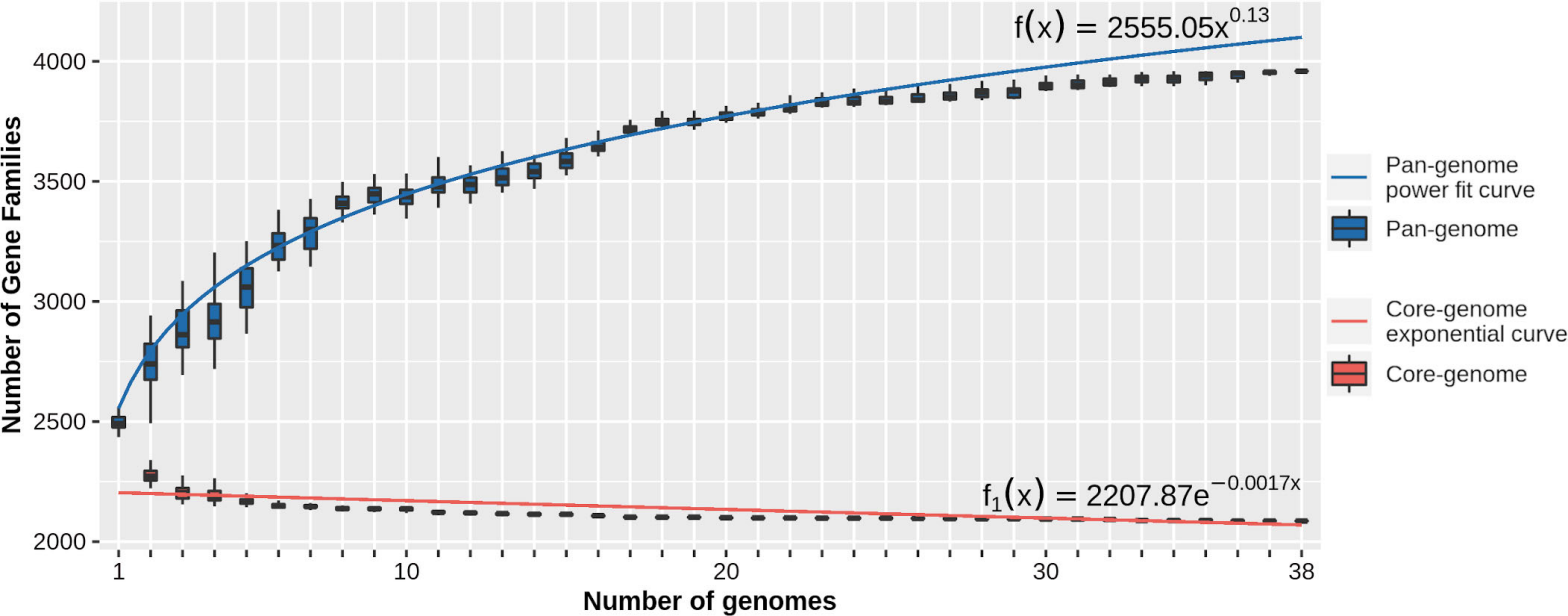
Pan-core plot depicting the trend of the pan-genome and the core-genome by the sequential addition of *S. aureus* strains to the analysis.

The sequential addition of *S. aureus* strains to the analysis highlighted a rapid decrease in size of the core-genome, as well as a continuous growth of the pan-genome. These trends revealed an “open” nature of the *S. aureus* pan-genome in agreement with the power curve, computed by the BPGA pipeline (**Figure 2**).

**Figure 3:**
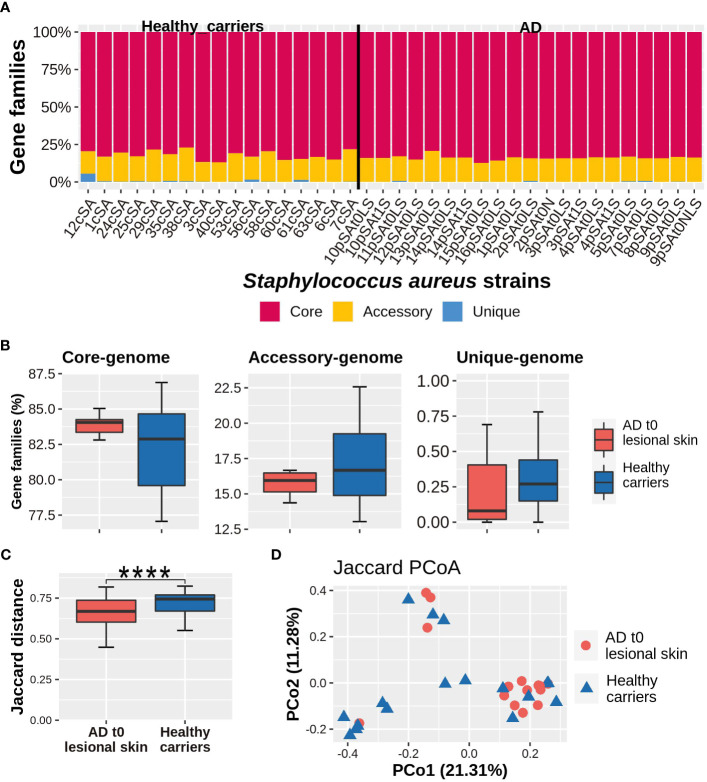
**(A)** Genomic composition of each *S. aureus* strain. **(B)** Relative abundance of the core, accessory, and unique genes. **(C)** Within-group diversity, measured by the Jaccard dissimilarity index. Values can range from 0 (i.e. the organisms share the same set of genes) to 1 (i.e. no genes in common). Comparisons between AD lesional strains and healthy carriers-related strains were carried out by Mann-Whitney *U*-test, considering a *p*-value ≤ 0.05 as statistically significant. (****p-value ≤ 0.0001). **(D)** Between-diversity analysis expressed by the Principal coordinate analysis, based on the Jaccard index and performed on AD lesional skin and healthy carriers-related strains. For each Principal coordinate, the variance explained is expressed by numbers within parentheses.

As shown in **Figure 3A**, the core-genome constituted the largest portion within the genome of each strain (median, IQR; 83.71%, 82.82%-84.24%), followed by the accessory genome (median, IQR; 16.14%, 15.13%-16.67%) and then by the unique genome (median, IQR; 0.16%, 0.05%-0.39%). No statistically significant differences were detected in the relative abundances of each component of the pan-genome, between the groups of strains isolated from the lesional skin of AD patients and those obtained from healthy subjects (**Figure 3B**). However, the healthy carrier strains were characterized by a significantly higher variability in the relative abundance of both core and accessory genes with respect to the strains coming from AD subjects (*p*-value_core_ = 0.013; *p*-value_accessory_ = 0.014).

The within-group genomic diversity, assessed on the basis of the Jaccard dissimilarity index (thus on the number of genes shared by each pair of strains), evidenced a higher within-group variability in the population of *S. aureus* strains isolated from healthy carriers than that obtained from AD (*p*-value < 0.0001) (**Figure 3C**). No significant partitions have been determined between the groups as evidenced by results obtained from principal coordinate analysis (PCoA), followed by the PERMANOVA test (**Figure 3D**). Furthermore, no genomic sequences have been exclusively found in either the AD-related or the healthy carriers-related strains.

#### 3.4.6 *bsa* operon and *dprA* gene search

The *bsa* operon encodes for a lantibiotic bacteriocin and it was detected in only two strains from each group. The aligned genes were characterized by sequence coverage >98% and sequence identity >85% with respect to reference sequences (**Table S6**).

On the other hand, virtually all strains (34/38) carried the *dprA* gene, known to affect the ability to assimilate foreign DNA (sequence coverage >99% and sequence identity >98%).

#### 3.4.7 Functional enrichment analysis

**Figure 4:**
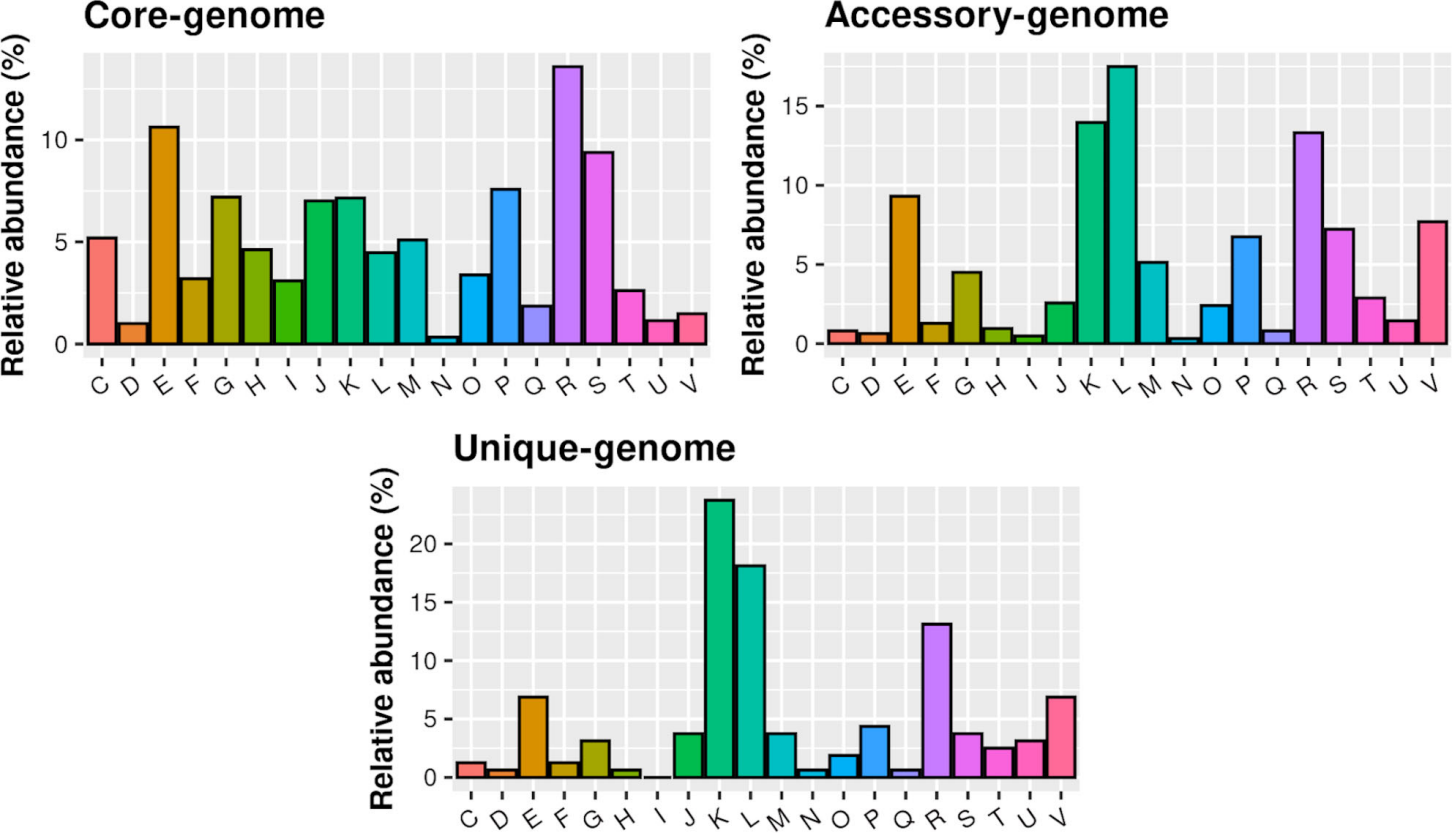
Functional distribution of the core, accessory and unique genes of the population, according to COG categories.

A functional enrichment analysis was performed, allowing the classification of the identified gene families according to the 20 COG categories. As shown in **Figure 4**, the most prevalent categories (>33%) in the core-genome were related to the Transport and metabolism of amino acids (E), nucleotides (F), carbohydrates (G), coenzymes (H), lipids (I), and inorganic ions (P). Transcription (K) and Replication, recombination and repair (L) mechanisms were the most enriched COG categories in the accessory- (13.96% and 17.49%, respectively) and in the unique-genome (23.73% and 18.11%, respectively). (**Figure 4**) General function prediction only (R) represents the top enriched COG category in both AD- (12.45%; 0.18%) and healthy carriers (12.28%; 0.29%) groups (**Figure S5**).

Interestingly, genes related to post-translational modification, protein turnover and chaperones (O), and intracellular trafficking, secretion, and vesicular transport (U) resulted in statistically more abundant among the AD lesional strains (*p*-value_O_ = 0.0039 and *p*-value_U_ = 0.043) (**Figure S5**).

### 3.5 AD strains are strong biofilm producers

Biofilm is an important factor for *S. aureus* skin colonization, and most of the isolates from AD lesional skin resulted in strong biofilm producers (Geoghegan et al., 2018). Accordingly, our results revealed that 67% (14/21) of AD strains were strong biofilm producers, 33% (7/21) were moderate biofilm producers, and no weak biofilm producers were found. In contrast, healthy subjects did not carry strong biofilm-producing strains, while moderate and weak biofilm producers accounted for 47% (8/17) and 53% (9/17) of the group, respectively. Statistically significant differences in the percentage of strong and weak biofilm producers were found between AD and healthy carrier strains (*p* value_strong_ ≤ 0.0001 and *p* value_weak_≤ 0.001).

### 3.6 Invasion ability could be not essential for AD strains

*S. aureus* is able to invade and survive in different host cells, including keratinocytes. To evaluate the invasive behavior of *S. aureus* strains from AD patients and healthy carriers, gentamicin protection assays on HaCat cells were performed. A significantly higher percentage of invasive *S. aureus* strains was observed among healthy carriers (88%) with respect to that recovered in AD patients (43%) (*p-*value ≤ 0.01).

## 4 Discussion

AD is one of the most frequent chronic inflammatory skin diseases characterized by recurrent eczematous lesions, itch and dry skin (David Boothe et al., 2017). The complex and multifactorial pathophysiology of AD involves a strong genetic predisposition, epidermal dysfunction, T-cell driven inflammation and skin microbiome abnormalities. In particular, *S. aureus* represents a dominant colonizer and pathogen, especially of the AD lesional sites, and it contributes to AD pathogenesis in many ways (e.g. skin barrier disruption and direct proinflammatory effects) (Geoghegan et al., 2018). However, the exact roles played by this microorganism *in vivo* are not completely understood.

Herein, we performed a genotypic and phenotypic characterization of patient-derived strains, sampled at two different stages of the disease, namely at acute phase before starting dupixent^®^ (dupilumab) therapy (t0) and at post-acute phase after reaching clinical remission with treatment (t1).

*S. aureus* was detected at t0 i) both in the anterior nares and on the lesional skin in 80% (12/15) of the patients and ii) on all of the sampled anatomical site in 40% (6/15), whereas iii) a cutaneous colonization alone was found in 20% (3/15) of the patients. In agreement with previous reports (Tauber et al., 2016; Totté et al., 2016), our results confirmed *S. aureus* as the predominant bacterial species on the inflamed skin of AD patients.

Following the dupilumab therapy, about 57% of patients still presented *S. aureus* colonization on the nose and 35.7% on the skin sites, while a complete eradication of *S. aureus* from all of the sampled body sites was observed in 28.57% of cases.

A significant positive correlation was found between the load of *S. aureus* on lesional skin and the severity of AD, defined by the objective SCORAD. *S. aureus* was more abundant in patients with moderate or severe disease (SCORAD ≥ 25). After the therapy, the significant improvement of the disease activity was accompanied by the simultaneous eradication of both nasal and skin colonizations, in 28% of AD patients. However, more than half of the AD patients were characterized by persisting *S. aureus* nasal colonization, confirming the anterior nares as the primary reservoir for *S. aureus* and as a major source for extra-nasal auto-transmission (Patel, 2001).

RAPD-PCR analysis on the AD-associated *S. aureus* isolates showed that in 80% of patients the nose, the lesional and the non-lesional skin, were colonized by the same strain during the flare, suggesting an intrapersonal spread of the same clone from one body site to another. The nasal isolates recovered at t1 resulted clonal to those sampled at t0 in 57% of patients, while the skin isolates were different from those sampled at t0 in 28% of patients, confirming the effectiveness of the treatment with dupilumab to reduce the *S. aureus*-induced inflammation.

Based on the intra-host genetic heterogeneity of *S. aureus*, we can also speculate about the presence of patient-specific strains that persist in the nose in the post-flare, constituting a potential reservoir for skin reinfections (Chiu et al., 2009). These findings should be carefully evaluated since they imply serious limitations in the long term efficacy of monoclonal antibodies-based therapeutic strategies

Specific therapies, targeting also the *S. aureus* nasal colonization, should be taken into consideration in this context.

Differently from the healthy controls *S. aureus* strains, the ones isolated from AD skin have been associated with higher levels of skin inflammation (Byrd et al., 2017 *).* Such findings evidence that the contribution of *S. aureus* to the complexity of the disease may be strain-dependent. Herein, we performed an *in silico* genotyping analysis on the *S. aureus* strains, evaluating their *spa-*types, *agr*-types and STs, to establish whether specific clones of *S. aureus* prevail among AD patients. The high degree of genetic heterogeneity in terms of *spa*-types and STs of our *S. aureus* population evidenced the absence of a prevailing genotype linked to AD. Analogously, the large genetic variability of *S. aureus* strains isolated from AD patients has been reported by previous studies (Capoluongo et al., 2001; Chung et al., 2008; Kim et al., 2009). *S. aureus* possesses a vast arsenal of VFs that facilitate its invasion into and its survival within the human host. The expression of these VFs is controlled by the *agr*-system (Bernabè et al., 2021). The *agr* operon in *S. aureus* includes *agrA*, *agrB*, *agrC*, and *agrD* genes, and it could be divided into four types (namely *agr-*I to *-*IV), according to the sequences of *agrC* and *agrD* genes. Analogously to previous works (Zhao et al., 2016; Nakamura et al., 2020; Tayebi et al., 2020), our findings highlighted that *agr*-I is the most predominant type among *S. aureus* strains, suggesting that *agr*-type is not associated with AD. As Nakamura and colleagues point out (Nakamura et al., 2020), the expression of a functional *agr* system is required for epidermal colonization and the induction of AD-like inflammation, suggesting a pivotal role of the *agr* virulence in the development of AD in humans. Thus, the attention should be focused on the retention of a functional *agr* system rather than on the sequence type of *agrC* and *agrD*.

VF produced by *S. aureus* have a crucial role in the inflammation process and in skin barrier dysfunction in AD (Taskapan and Kumar, 2000; Tomi et al., 2005; Syed et al., 2015; Geoghegan et al., 2018). Sequence alignment between our strains and the reference database evidenced that most of the detected VF genes were widely spread in the population (prevalence ≥ 50%). In particular, all VFs involved in the immune evasion, adhesion, and toxic activity (exoenzymes and toxins) were found in the whole population, suggesting a common pathogenic potentiality that is independent from pathophysiological context. Aziz and colleagues suggest that specific enterotoxins may play a role in AD (Aziz et al., 2020). Instead, we found that genes encoding for enterotoxins were significantly more prevalent among healthy carrier strains. Taken together, these evidences may suggest that the role played by enterotoxins is not fundamental to *S. aureus* in AD. Overall, our results are consistent with previous studies, in which no CCs, *agr-*types, and VFs have been consistently linked to the pathogenesis of infection or colonization in AD (Benito et al., 2016; Fleury et al., 2017; Geoghegan et al., 2018).

Both genotypic and phenotypic analysis were performed in our study in order to assess the antibiotic resistance profile of all the 38 *S. aureus* strains. The WGS analysis evidenced the presence of a discrete collection of AMR genes, most of which were ubiquitously found in the bacterial population. Regardless of isolation origin, a widespread susceptibility to several antibiotics was revealed by the phenotypic analysis. Only methicillin-susceptible *S. aureus* strains were detected in the population, according to both of the analyses. Taken together, these results highlighted an overall agreement between the WGS-predicted resistance and phenotypic susceptibility tests, demonstrating a high concordance to 10 different agents (gentamicin, fusidic acid, mupirocin, linezolid, vancomycin, erythromycin, clindamycin, benzylpenicillin, oxacillin and teicoplanin), with some exceptions. According to the phenotypic test, the *S. aureus* strain 7pSAt0LS was benzylpenicillin-susceptible, although it carried the *blaZ* gene. As previously reported (Ivanovic et al., 2023), we could hypothesize that incompleteness of the *bla* operon could be responsible for the susceptible phenotype of this strain. No corresponding AMR genes were found in some strains showing an intermediate or resistant phenotype to clindamycin, benzylpenicillin, erythromycin, teicoplanin and oxacillin. However, we may reasonably assume that these intermediate or resistant phenotypes are likely mediated by AMR genes not yet determined, due to the current limited repertoire of sequences available to date. Phenotypic tests revealed that all the strains were tetracycline-susceptible, although tet38 and its regulator mgrA were ubiquitously found in the population. This apparent incongruence may be explained by the study conducted by Ding and colleagues (Ding et al., 2008), that revealed a distinct expression profile of the *S. aureus* tet38 efflux pump between the *in vitro* growth model and the *in vivo* infection model in mice, hypothesizing that environmental triggers, other than antimicrobial substances, can play an important role in the overexpression of tet38. Successively, the authors (Chen and Hooper, 2018) evaluated whether the selective overexpression of tet38 has an effect on response of *S. aureus* to tetracycline in the *in vitro* and *in vivo* models, observing a reduced efficacy of the antibiotic only in the abscess (*in vivo* model).

In our study, we could not assess the concordance between genotypic and phenotypic profiles to several antibiotics (i.e. levofloxacin, rifampicin, tigecycline, daptomycin and trimethoprim/sulfamethoxazole), whose resistance is due to mutations on specific genes. Further studies will be conducted to investigate the presence of these genetic mutations. Although genomic analysis represents a promising tool for the determination of the resistance profile of microbial strains, our findings evidence that the phenotypic characterization of strains still plays a primary role in the evaluation of the antimicrobial susceptibility.

A considerable degree of variety within the *S. aureus* population was demonstrated by the results of the reconstruction of phylogenetic relationships among strains. It is likely that the diversity pattern does not reflect the natural division of the species into subgroups and, in this context, belonging to a certain *spa* or MLST type appears to be the most appropriate trait explaining the genetic clustering of strains. The availability of a number of putative non-mutually exclusive mechanisms that might contribute to the patterns seen in the data could be used to explain these findings. First, there might be considerable biological obstacles preventing genetic exchange between lineages of the *S. aureus* population; these obstacles include restriction-modification (RM) systems, which operate as a major roadblock to DNA transfer (Corvaglia et al., 2010). The genetic exchange may be promoted or prevented by several systems that restrict phage and plasmid infection, such as CRISPR-cas systems (Marraffini and Sontheimer, 2008), phage-control systems (Ram et al., 2012; Ram et al., 2014), and variety in cell surface phage receptors (Winstel et al., 2014; Li et al., 2015). Other mechanisms, including plasmid incompatibility, functional restraints, or even functional redundancy of genes, can strengthen these genetic barriers and restrict the maintenance of DNA sequences acquired by clones. The existence of barriers across lineages also increases the chance that within each of these subgroups, recombination events may be responsible for the observed genetic similarity among strains. Another idea is that the existing lineages may be the isolated offspring of several extinct failed lineages, thus the consequence of the formation of a few extremely successful groups.

Locally, it is frequently noted that only a small number of CCs or STs dominate at any given time, but this pattern appears to be broken by the invasion of new dominant clones (Blanc et al., 2007; Chambers and Deleo, 2009), demonstrating that different clones coexist and compete for the same niche(s) (Sakwinska et al., 2009). In the *S. aureus* literature, examples of epidemic strain invasion and replacement are still accumulating (Uhlemann et al., 2014).

The Pangenome analysis of the *S. aureus* population revealed that, in each strain, approximately half of the pangenome has been represented by core genes. Although we expect that this evidence could be influenced by the limited sample size of the analyzed population, it is to note that the obtained results underline a very high degree of shared genetic sequence among the *S. aureus* population. A possible explanation to these findings is represented by the main role that the horizontal gene transfer plays among *S. aureus* strains, together with high rates of mobile genetic elements exchange during their co-colonization (McCarthy et al., 2014). According to previous observations, the core-genome of the *S.aureus* population is impacted by core genome transfer (CGT) supported by broad- and fine-scale trends in homoplasy attributable to macro-recombination, most likely driven by mobile elements (Everitt et al., 2014). The high degree with which the sequences are acquired and preserved in the genome of *S. aureus* leads to the formation of a gene reservoir which allows the single strains to colonize and adapt to a large variety of ecological niches, as demonstrated by the majority of cases, where the same clone predominates in inflamed and non-inflamed tissue. In this context, however, it should be highlighted that exposure to inflammatory conditions, such as those present in the skin lesions of AD subjects, is associated with a significant reduction in the variability in the gene content of the *S. aureus* pangenome, in line with the results obtained from the present study. On one hand, this reduction indicates the inflammatory conditions act to optimize the genetic content of the strains through selective pressure. On the other hand, it allows us to speculate about the presence of a certain degree of functional redundancy within the genome of the species.

Functional enrichment analysis showed that genes related to post-translational modifications (PTMs), protein turnover and chaperones, intracellular trafficking, secretion and vesicular transport resulted statistically more abundant among the AD strains. Although few studies have been conducted so far, evidence suggests that PTM mechanisms in *S. aureus* may be involved in the pathogenic lifestyle, as well as, in other cellular processes. For instance, glycosylation is involved in the cell wall teichoic acid (WTA) biosynthesis pathway; WTAs play an important role in the bacterial physiology, resistance to antimicrobial molecules, host interaction, virulence and biofilm formation (Mistretta et al., 2019). Likewise, phosphorylation is a common PTM mechanism, that is involved in the central metabolic processes and in the regulation of the efflux-pump NorA, by acting on the MgrA protein; however, the role of phosphorylation in staphylococcal pathogenesis, as well as the role of other PTMs, needs to be further investigated, especially regarding AD (Ohlsen and Donat, 2010). Moreover, bacteria are able to manipulate the host immune response through the secretion of membrane vesicles, that play an essential role in the delivery of bacterial effectors promoting virulence, biofilm formation, signal transduction, cytotoxicity, resistance to antimicrobial skin fatty acids and in skin inflammatory disorders (Gurung et al., 2011; Alpdundar Bulut et al., 2020; Kengmo Tchoupa and Peschel, 2020; Staudenmaier et al., 2022). Several studies have demonstrated the importance of staphylococcal biofilms in the pathogenesis of AD (Allen et al., 2014; Sonesson et al., 2017; Di Domenico et al., 2018; Haasbroek et al., 2022). Most of our AD strains were strong biofilm producers, confirming the importance of biofilm in i) the increased resistance to the host immune responses, in ii) the reduced susceptibility to antimicrobials and in iii) inflammation, thus, promoting skin colonization and persistence. Previous works have shown that *S. aureus* can invade host cells and persist intracellularly in cell culture models, including keratinocytes (Lowy, 2000; Sendi and Proctor, 2009; Sayedyahossein et al., 2015). Our results revealed that only a limited number of AD strains were invasive if compared to healthy carriers, suggesting that the ability to invade could be not essential for AD strains. Overall results evidence that genotypic analysis fails to identify specific features associable to AD strains and partially this may be due to the relatively low number of strains analyzed. However, the phenotypic characterization highlights substantial differences between the two groups regarding biofilm production and invasiveness of strains.

### 4.1 Conclusion

Although phenotypic differences were found between AD and healthy carrier *S. aureus* strains, our results evidenced no predominant genotypic traits in AD strains, suggesting the absence of *S. aureus* clones shared among AD patients.

We could speculate that the functional role played by *S. aureus* in AD likely depends on other factors, such as a differential gene expression pattern on the AD skin sites or PTM mechanisms that are involved in the *S. aureus* pathogenic lifestyle.

## Data availability statement

The data analyzed in this study is subject to the following licenses/restrictions: Original data will be available on request. Requests to access these datasets should be directed to francesca.brunetti@uniroma1.it. The data presented in the study are deposited in the DDBJ/ENA/GenBank repositories, accession numbers:JARZEJ000000000, JARZEK000000000, JARZEL000000000, JARZEM000000000, JARZEN000000000, JARZEO000000000, JARZEP000000000, JARZEQ000000000, JARZER000000000, JARZES000000000, JARZET000000000, JARZEU000000000, JARZEV000000000, JARZEW000000000, JARZEX000000000, JARZEY000000000, JARZEZ000000000, JARZFA000000000, JARZFB000000000, JARZFC000000000, JARZFD000000000, JARZFE000000000, JARZFF000000000, JARZFG000000000, JARZFH000000000, JARZFI000000000, JARZFJ000000000, JARZFK000000000, JARZFL000000000, JARZFM000000000, JARZFN000000000, JARZFO000000000, JARZFP000000000, JARZFQ000000000, JARZFR000000000, JARZFS000000000, JARZFT000000000, JARZFU000000000.

## Ethics statement

The studies involving human participants were reviewed and approved by Ethics Committee of Sapienza University of Rome. The patients/participants provided their written informed consent to participate in this study.

## Author contributions

MPC, ATP and SG conceived and supervised the study. SG enrolled the patients and collected the samples. ALC, LM and CL analyzed the samples and performed the experiments. GR performed bacterial identification and the antibiotic susceptibility tests. FB and MM performed bioinformatic and statistical analysis. ALC and FB wrote the draft manuscript. ALC, FB, MPC and MM wrote and reviewed the manuscript. All authors contributed to the article and approved the submitted version.
