# Supplementary figures and images for: Atopic dermatitis-derived Staphylococcus aureus strains: what makes them special in the interplay with the host
Source: Front Cell Infect Microbiol. 2023 Jun 14;13:1194254. doi: 10.3389/fcimb.2023.1194254 (PMC10303148; doi:10.3389/fcimb.2023.1194254)

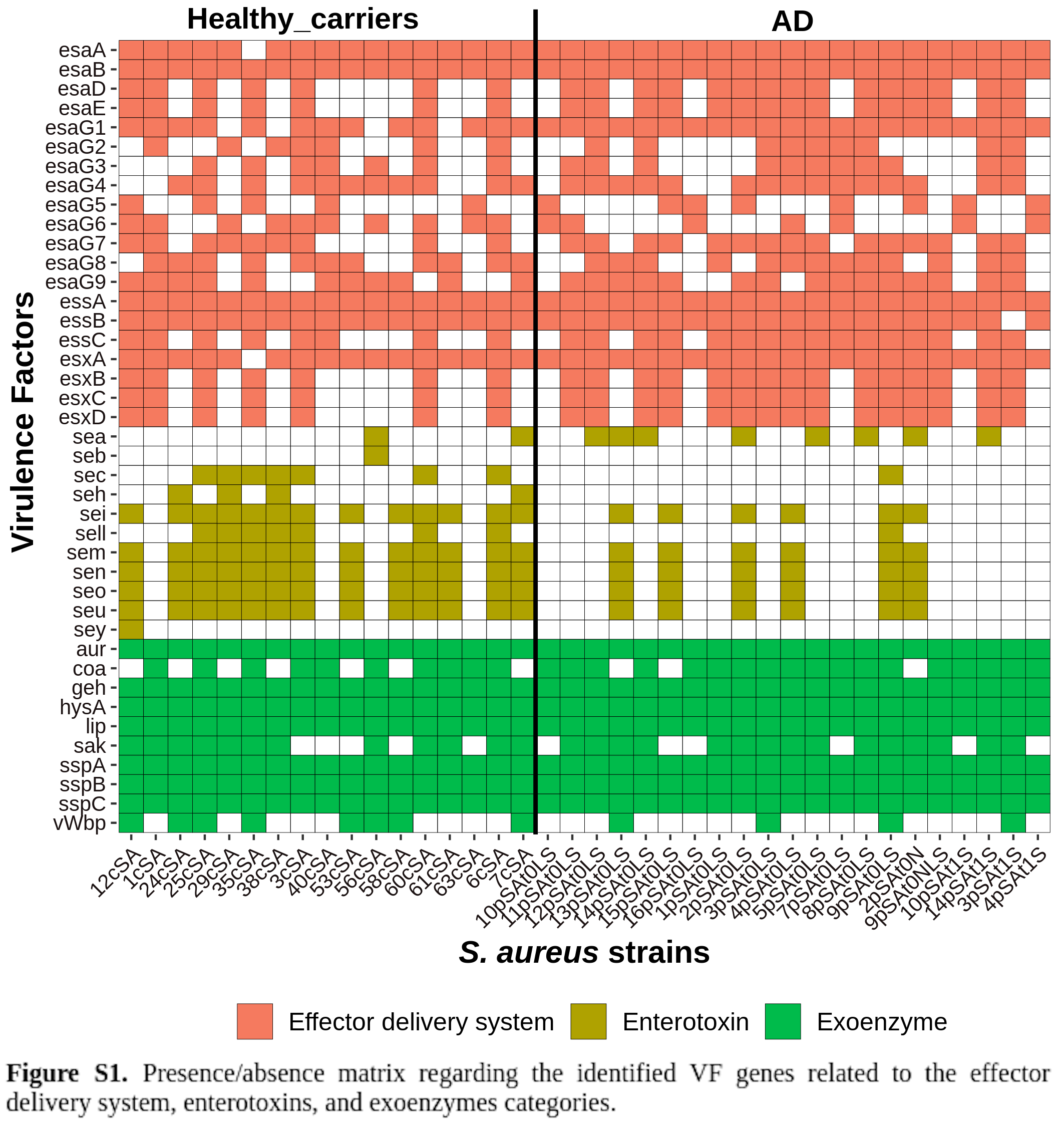

Supplement: Supplementary file 1 [file Image_1.tiff]

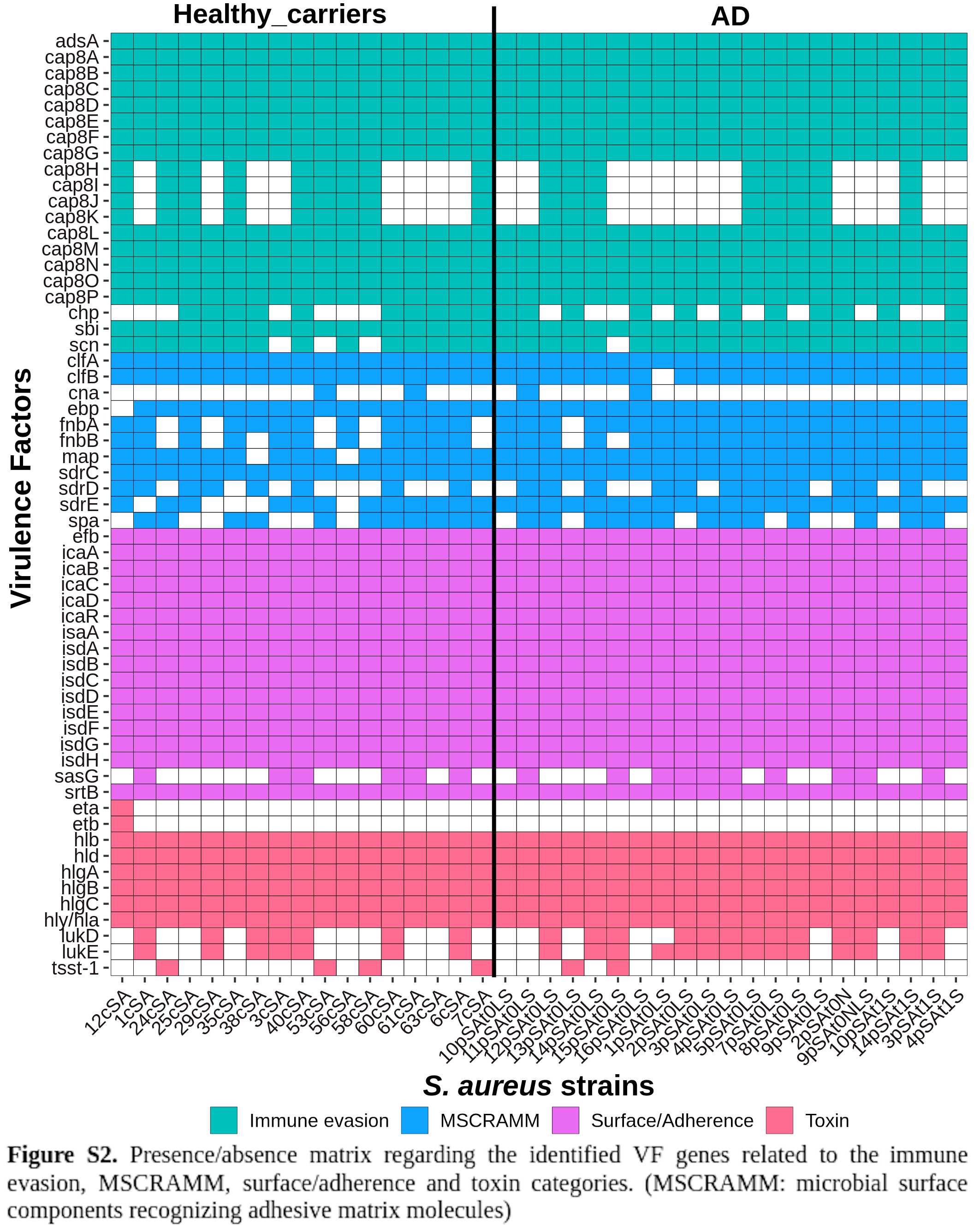

Supplement: Supplementary file 2 [file Image_2.jpeg]

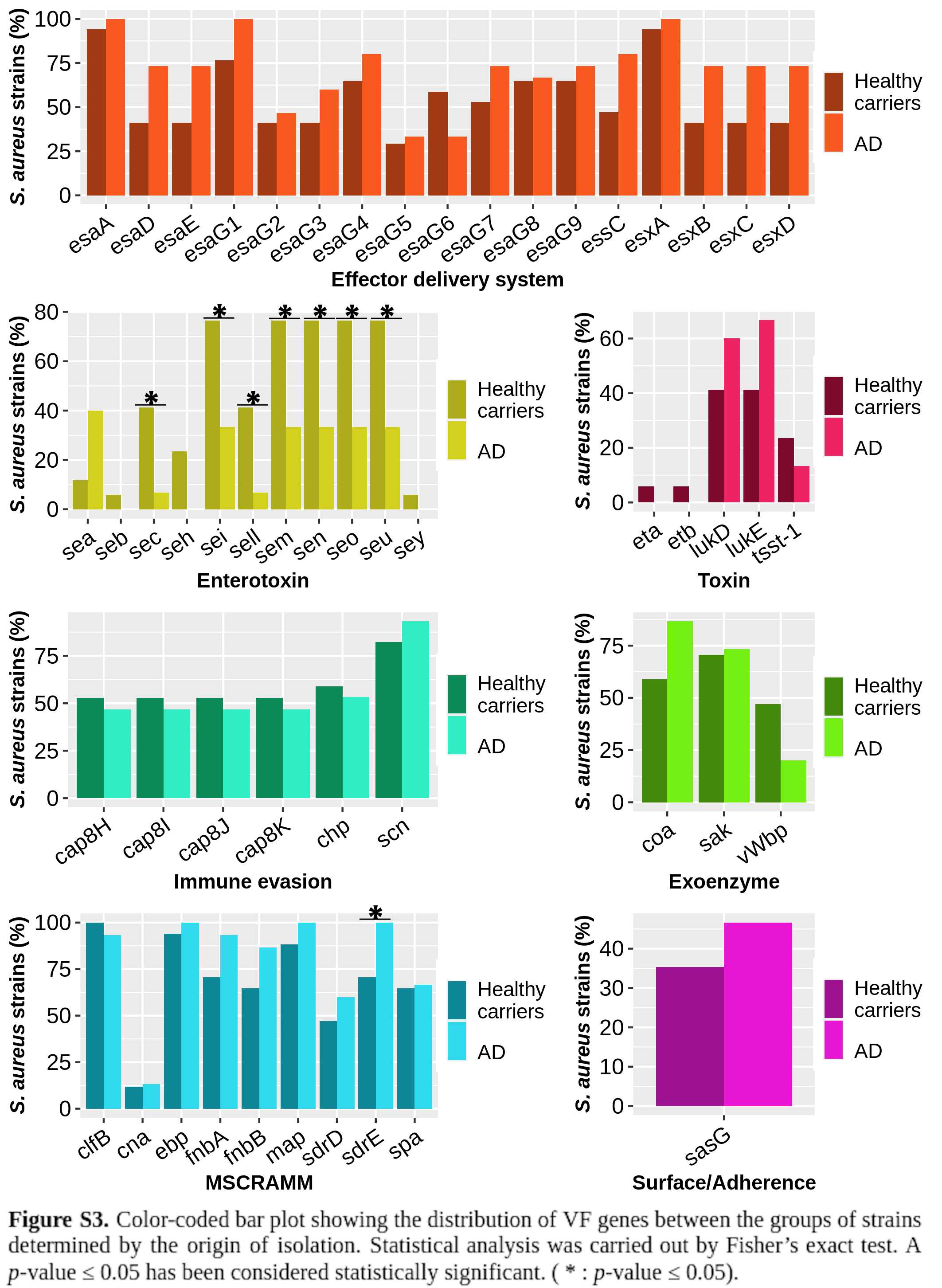

Supplement: Supplementary file 3 [file Image_3.jpeg]

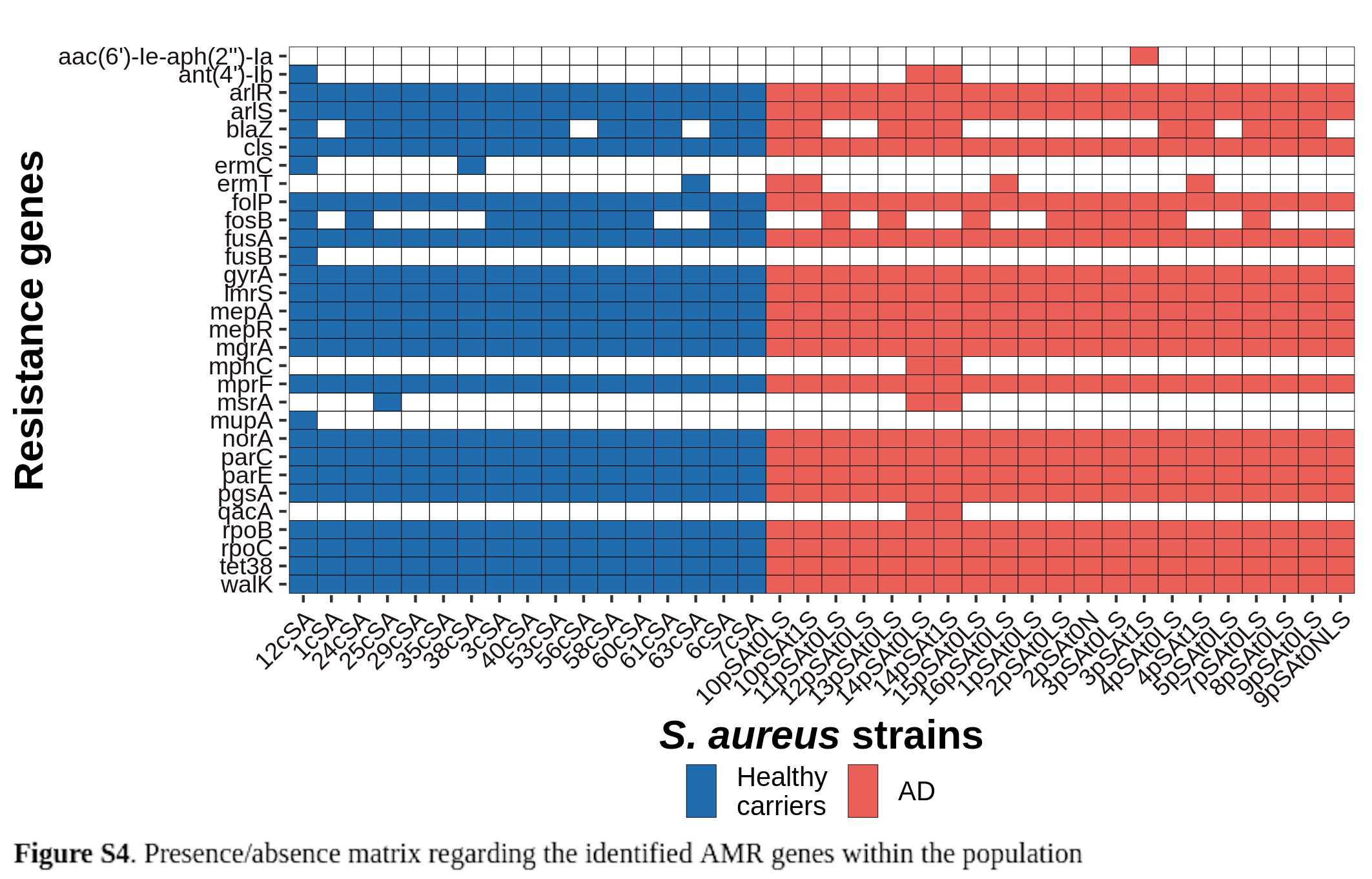

Supplement: Supplementary file 4 [file Image_4.jpeg]

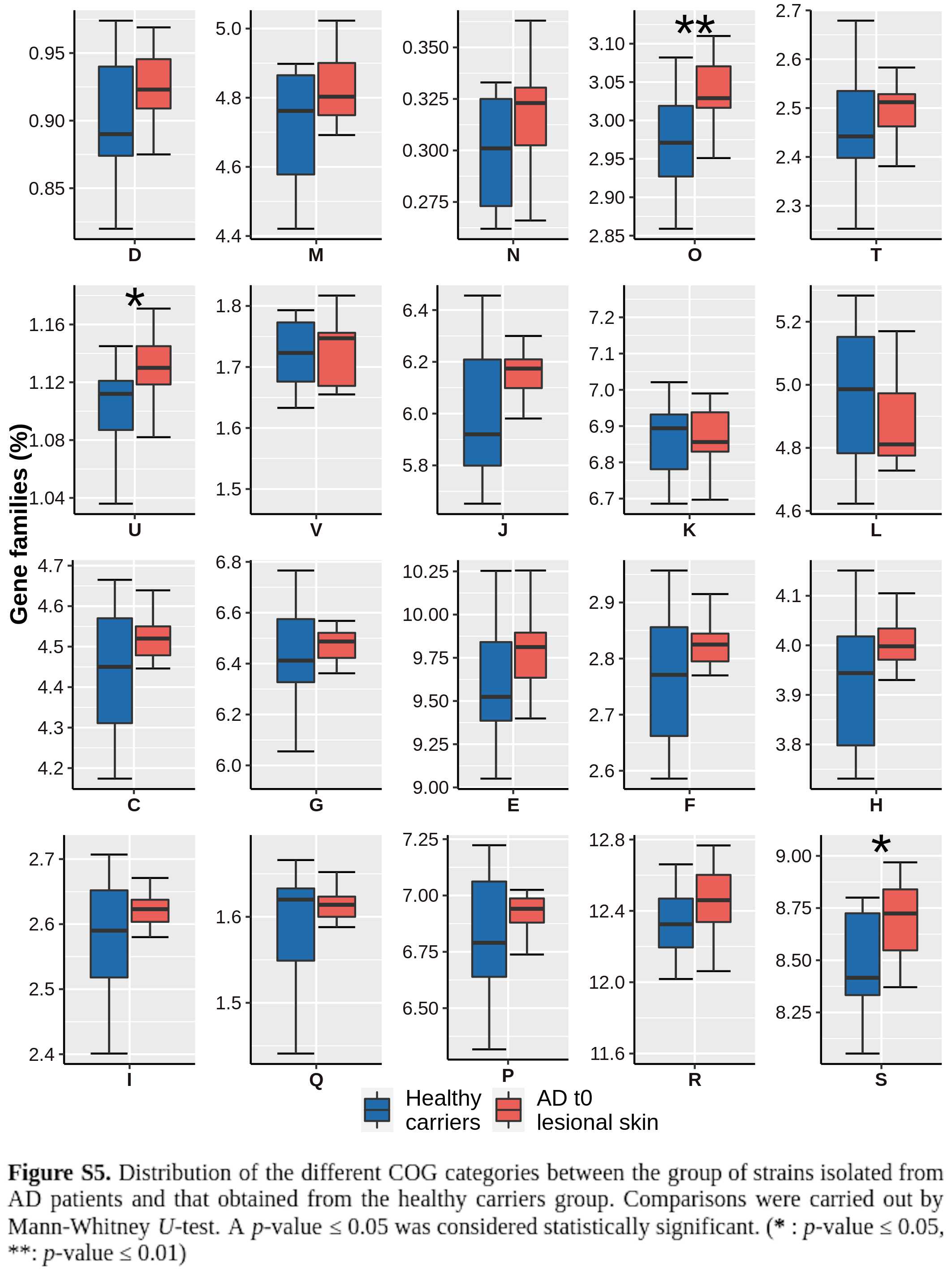

Supplement: Supplementary file 5 [file Image_5.jpeg]
